# Can glucagon-like peptide-1 receptor agonists cause acute kidney injury? An analytical study based on post-marketing approval pharmacovigilance data

**DOI:** 10.3389/fendo.2022.1032199

**Published:** 2022-12-13

**Authors:** Shichao Dong, Chuan Sun

**Affiliations:** ^1^ Department of Pharmacy, The Second Hospital of Tianjin Medical University, Tianjin, China; ^2^ Department of Pharmacy, Children’s Hospital of Nanjing Medical University, Nanjing, China

**Keywords:** glucagon-like peptide-1 receptor agonist, acute kidney injury, adverse event reporting system, data mining, onset time, outcome

## Abstract

Clinical studies after marketing have shown that the use of glucagon-like peptide-1 receptor agonist(GLP-1RA) may lead to acute kidney injury(AKI). However, few epidemiological studies have investigated the risk, clinical features, and outcomes of AKI caused by different GLP-1RA. In this study, Adverse Event Reporting System (FAERS) data were used to compare the association between different GLP-1RA and AKI in the real world.

**Methods:**

FAERS data from January 2004 to December 2021 were mined using disproportionality analysis and Bayesian analysis to determine the correlation between different GLP-1RA and AKI, and the onset time, mortality, and hospitalization rate of different GLP-1RA were analyzed.

**Results:**

We identified 2670 cases of AKI events associated with GLP-1RA, of which liraglutide was the most commonly reported (34.98%). The patients with AKI were mainly males (47.94%), and the age group was mainly 45-84 years old (73.15%). obese patients with weight more than 99kg (24.42%) were more likely to have AKI. According to different signal mining methods, reporting odds ratio (ROR) (1.50, 95% confidence interval =1.41-1.60) and Bayesian confidence Propagation neural network (0.57, 95% confidence interval =0.54), liraglutide was more strongly associated with AKI than other GLP-1RA. The median time to onset of AKI was 63 days [quartile range (IQR): 15-458.5 days]. In addition, the hospitalization rate and fatality rate of patients with GLP-1RA-related AKI were 45.28% and 4.23% respectively.

**Conclusions:**

Based on the data in the FAERS database, we analyzed the risk, onset time, and adverse reaction outcomes of GLP-1RA-induced AKI in detail. The results showed that liraglutide had the highest risk of AKI. From the early stage of treatment, we need to monitor patients’ renal function regularly, especially for patients with high kidney risks such as obesity and age.

## Introduction

Glucagon-like peptide-1 receptor agonist(GLP-1RA) is a new type of hypoglycemic drug, the application of which is derived from the incretin effect. The incretin effect is a phenomenon, which shows that the insulin response caused by oral glucose is much larger than that caused by intravenous glucose infusion (1, 2). Therefore, glucagon-like peptide-1 (GLP-1) and Glucose-dependent insulinotropic polypeptide (GIP) were found. These two intestinal peptides secreted after nutrient intake were used to explain this phenomenon. When people eat, GLP-1 increases insulin secretion in response to glucose intake in the body and inhibits glucagon secretion of islet cells, resulting in a decrease in postprandial blood glucose levels (3). Current studies have shown that patients with pre-diabetes or type 2 diabetes mellitus often have defects in the effect of incretin, while exogenous GIP does not show a good insulin-promoting effect (4). Therefore, more and more GLP-1RA have been put into the market and show superior hypoglycemic effect. Up to now, GLP-1RA has been approved by FDA for marketing including liraglutide, dulaglutide, semaglutide, lixisenatide, albiglutide, and exenatide. According to different chemical structures, GLP-1RA can be divided into incretin-mimetics (exendin-4 analogs, such as daily exenatide, weekly exenatide, and lixisenatide) and human GLP-1RA (such as liraglutide, dulaglutide, semaglutide, and albiglutide) (3). A series of clinical trials, such as LEADER (5), SUSTAIN 6 (6), HARMONY (7), REWIND (8), and PIONEER 6 (9), proved that GLP-1RA could reduce the risk of cardiovascular events in addition to the effect of blood glucose reduction. In addition, GLP-1RA, including liraglutide (10), dulaglutide (8), and exenatide (11), has shown renal benefits in clinical trials.

Although GLP-1RA shows advantages in reducing glucose and weight (12), adverse reactions can not be ignored. Studies have shown that the most common side effects of GLP-1RA are from the gastrointestinal tract, including nausea, vomiting, diarrhea, and constipation (13). Furthermore, Pancreatitis, thyroid cancer, and cholelithiasis have also raised concerns about their use. Although several retrospective studies on GLP-1RA have been carried out, the results of the above adverse reactions are contradictory. Therefore, the safety of this kind of drug needs longer clinical observation (14). Interestingly, although GLP-1RA significantly reduced the risk of combined renal endpoints, including new proteinuria and persistent elevation of eGFR, in the clinical trial of GLP-1RA, the use of semaglutide was associated with a higher risk of AKI compared with placebo, even though the value is not statistically significant (15, 16). Another study on patients with type 1 diabetes showed that liraglutide could make positive changes in the levels of kidney sensitive biomarkers, and GLP-1RA may have a protective effect on patients with early renal damage in type 1 diabetes (17). However, it cannot be considered that GLP-1RA is absolutely safe for patients with renal risk. Nearly 80 post-marketing reports of exenatide show that patients have acute renal failure or renal insufficiency after medication (18), and 95% of cases are accompanied by renal risk factors, including the use of nephrotoxic drugs, hypertension, heart failure, etc. At the beginning of the marketing of liraglutide and semaglutide, some cases also reported AKI (18, 19), interstitial nephritis, and acute tubular necrosis (20, 21). The renal function of these patients did not completely recover after drug withdrawal, and the renal function and urinary protein were not improved. Despite these renal adverse outcomes, GLP-1RA is considered safe, including in patients with chronic kidney disease (22).

Compared with other common adverse reactions, the risk of AKI with GLP-1RA is not significant, which makes it easier for people to ignore its adverse effects. Currently, most of the warning information about GLP-1RA comes from clinical trials or case reports, and only a limited number of pharmacovigilance studies have explored other adverse reactions of this kind of drug (23, 24). No study has systematically analyzed the risk and characteristics of GLP-1RA-related AKI, and its clinical characteristics in the real world are blank. The inconsistent results for the kidney suggest that more studies are needed to assess the association between GLP-1RA and AKI. Hence, we believe it is necessary to mine the pharmacovigilance database [FDA Adverse Event Reporting System (FAERS) was selected as the target in this study] to compare the correlation between different GLP-1RA and AKI in the real world, and to analyze the general clinical characteristics of this adverse reaction, so as to improve clinical medication safety.

## Materials and methods

### Data source

**Figure 1:**
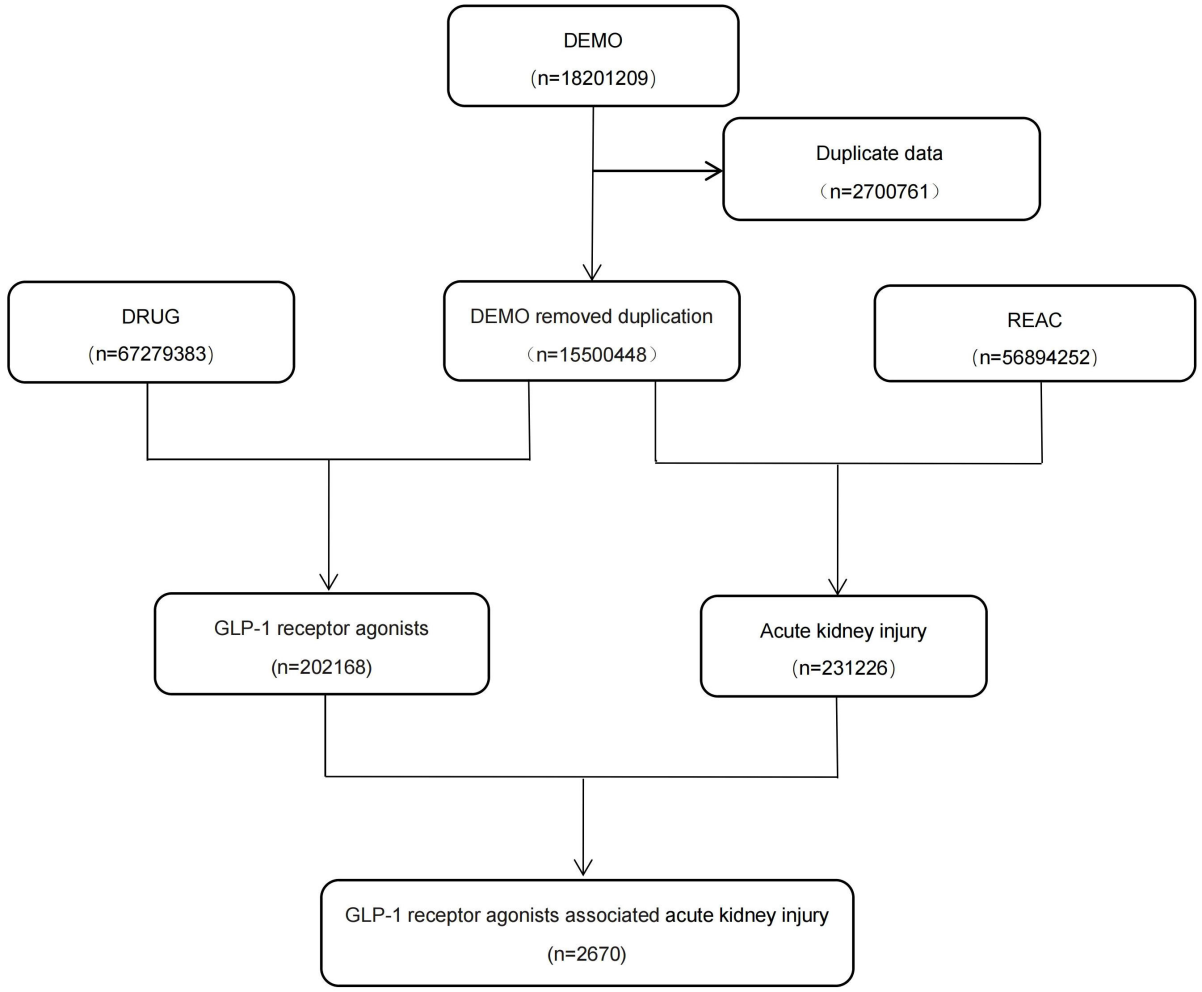
Process of screening cases of acute kidney injury caused by GLP-1 receptor agonists from FAERS database. FAERS, FDA Adverse Event Reporting System; GLP,glucagon-like peptide-1.

FAERS is not only an open database but also a voluntary reporting system. Medical professionals, consumers, and drug manufacturers can report adverse drug information to FAERS (25). The incidents reported by FAERS consist of seven files, Including DEMO (Patient Demographics and Administrative Information), DRUG (DRUG/Biologic Information), REAC (all terms coded for the Event), OUTC (patient outcomes), RPSR (Report Sources), THER (Drug therapy start dates and end dates), INDI (all terms coded For the indications. In this study, adverse event information from January 2004 to December 2021 in the FAERS system was retrospectively analyzed, and a total of 1,8201,209 reports were obtained. After deleting duplicate information (according to FDA’s recommendation, the latest FDA_DT was selected when CASE_ID was the same as FDA_DT), We ended up with 1,5500,448 actual reports (**Figure 1**).

### Data cleaning

In this study, the database was searched with the preferred term (PT) in the medical dictionary for regulatory activities (MedDRA 25.0) as the name of adverse reactions. The keywords related to AKI are acute kidney injury, oliguria, anuria, blood creatinine increased, blood urea increased, nephropathy toxic, tubulointerstitial nephritis, tubular obstruction, acute tubular necrosis, acute renal injury, pre-renal failure, acute renal failure, dialysis, hemodialysis, peritoneal dialysis. Furthermore, we query MICROMEDEX (Index Nominum) to determine the generic name and brand name of GLP-1RA (**Table 1**).

**Table 1:** Information summary of GLP-1 receptor agonists.

Generic Name	Brand name	Approval year
Exenatide	BYDUREON BCISE, BYDUREON, BYETTA, BAIETTA	2005
Liraglutide(Insulin Degludec/Liraglutide)	VICTOZA, SAXENDA, XULTOPHY	2010
Dulaglutide	TRULICITY	2014
Albiglutide	TANZEUM, EPERZAN	2014
Lixisenatide(Insulin Glargine,Recombinant/Lixisenatide)	ADLYXIN, LYXUMIA, SOLIQUA	2016
Semaglutide	OZEMPIC, WEGOVY, RYBELSUS	2017

### Data mining

Disproportionality analysis is the mainstream signal detection method in the world (26). Based on the principle of disproportionality analysis, it can be divided into reporting odds Ratio (ROR) and proportional reporting ratio (PRR) according to the difference in algorithms. Bayesian theory is introduced on the basis of disproportionality analysis, Thus, Bayesian confidence propagation neural network (BCPNN) and multi-item gamma Poisson shrinker (MGPS) are obtained (24).In this study, the four methods mentioned above were used to mine the target signals (**Table 2**), to compare whether different GLP-1RA is associated with AKI. It is worth noting that, to eliminate interference, only GLP-1RA monotherapy was included in this study, that is, primary suspect DRUG (PS) was screened out under the ROLE_COD project of the DRUG file, and ensure that drugs labeled Secondary Suspected drug(SS) and Concomitant drugs(C) corresponding to no other GLP-1RA. Furthermore, we evaluated the occurrence time of adverse reactions of different GLP-1RAs and determined the specific time by the time interval between the starting time of medication (START_DT) and the occurrence time of adverse events (EVENT_DT). This item excludes the illogical record that EVENT_DT precedes START_DT. Finally, we analyzed the outcome of adverse events, screened out the reports with results from all events, and calculated the probability of serious events such as death and hospitalization in the overall event.

**Table 2:** Data mining methods and specific formulas.

Algorithms	Equation	Criteria
ROR	ROR= (a/c)/(b/d) 95%CI=e^ln(ROR) ± 1.96(1/a + 1/b + 1/c + 1/d)^0.5^	95%CI > 1 N≥2
PRR	PRR= (a/(a+b))/(c/(c+d)) χ^2^=Σ((O-E)2/E);(O=a,E=(a+b)(a+c)/(a+b+c+d)	PRR≥2 χ^2^≥4, N≥3
BCPNN	IC=log_2_a(a+b+c+d)/((a+c)(a+b)) IC025=e^ln(IC) - 1.96(1/a + 1/b + 1/c + 1/d)^0.5^	IC025>0
MGPS	EBGM=a(a+b+c+d)/((a+c)(a+b)) EBGM05=e^ln(EBGM) - 1.64(1/a + 1/b + 1/c + 1/d)^0.5^	EBGM05>2 N>0

BCPNN, Bayesian confidence propagation neural network; CI, confidence interval; EBGM, empirical Bayesian geometric mean; EBGM05, the lower 90% one‐sided CI of EBGM; IC, information component; IC025, the lower limit of the 95% two‐sided CI of the IC; MGPS, multi‐item gamma Poisson shrinker; N, the number of co‐occurrences; PRR, proportional reporting ratio; ROR, reporting odds ratio; χ 2, chi‐squared; a, number of reports containing both the suspect drug and the suspect adverse drug reaction; b, number of reports containing the suspect drug with other adverse drug reactions (except the event of interest); c, number of reports containing the suspect adverse drug reaction with other medications (except the drug of interest); d, number of reports containing other medications and other adverse drug reactions.

### Statistical analysis

Descriptive analysis was used to demonstrate the clinical features of GLP-1RA-associated AKI as a result of this study. In this study, a nonparametric test (Mann-Whitney test was used in two groups, and Kruskal-Wallis test was used in multiple groups) was used to compare the onset time of AKI caused by different GLP-1RA.In this study, a nonparametric test (Mann-Whitney test for two groups and Kruskal-Wallis test for multiple groups) was used to compare the onset time of AKI after using different GLP-1RA. Fisher’s Exact test or Pearson’s Chi-Square test was used to comparing the adverse reactions of GLP-1RA.P<0.05 with 95% confidence intervals was considered statistically significant. SAS, Version 9.4 (SAS Institute Inc.) was used for statistical analysis and data mining.

## Result

### Descriptive analysis

From January 2004 to December 2021, 202168 reports related to GLP-1RA were found in FAERS, and 231226 reports caused AKI. Finally, we obtained 2670 reports of AKI mediated by GLP-1RA. Among these reports, the most suspected liraglutide was reported (n= 934,34.98%), and the least was lixisenatide, with only 4 reports (0.15%) related to AKI. Most reports were reported by North America (1676, 62.77%), followed by Europe (816, 30.56%), and were mainly reported by health professionals (1416, 53.03%). Males were more prone to AKI than females (47.94% vs 43.48%), and the age group was mainly 45-84 years old (73.15%). The average age of male patients was 62.09 ± 11.22, and the average age of female patients was 60.71 ± 11.19. Overweight patients (> 99kg, 24.42%) were the main group with AKI. In addition, AKI was more common in the group diagnosed with type 2 diabetes mellitus (34.57%), followed by diabetes patients without classification (21.99%). The specific clinical features of AKI caused by GLP-1RA are shown in **Table 3**.

**Table 3:** The specific clinical characteristics of GLP-1RA induced acute renal injury were excavated from FAERS.

Characteristics	Reports, No. (%)
Gender	
Male	1280 (47.94)
Female	1161 (43.48)
Unknown	229 (8.58)
Age (year)	
0-14	9 (0.34)
15-44	155 (5.81)
45-64	1046 (39.18)
65-84	907 (33.97)
>84	15 (0.56)
Unknown	538 (20.15)
Weight (kg)	
0-64	97 (3.63)
65-99	629 (23.56)
>99	652 (24.42)
Unknown	1292 (48.39)
Reporting area	
Oceania	29 (1.09)
Europe	816 (30.56)
South America	17 (0.64)
North America	1676 (62.77)
Asia	86 (3.22)
Africa	1 (0.04)
Unknown	45 (1.69)
Reporter	
Customer	752 (28.16)
Professional medical	1416 (53.03)
Other Reporters	492 (18.43)
Suspected drugs	
Exenatide	880 (32.96)
Liraglutide	934 (34.98)
Dulaglutide	546 (20.45)
Albiglutide	62 (2.32)
Lixisenatide	4 (0.15)
Semaglutide	244 (9.14)
Indications	
Diabetes mellitus	587 (21.99)
Type 2 diabetes mellitus	923 (34.57)
hyperglycemia	8 (0.3)
Abnormal blood glucose	3 (0.11)
Type 1 diabetes mellitus	9 (0.34)
Impaired glucose tolerance	4 (0.15)
Poor glycemic control	7 (0.26)
Non-insulin-dependent Diabetes mellitus	67 (2.51)
insulin resistance	4 (0.15)
Unknown	1058 (39.63)

### Data mining results

We apply four methods to process the target signal and detect it according to the criteria corresponding to each algorithm. As shown in **Table 4**, liraglutide had the strongest association with AKI by ROR and BCPNN, followed by semaglutide and lixisenatide. Exenatide, dulaglutide, and albiglutide did not meet the test criteria, so the three drugs mentioned above were not shown to be associated with AKI.

**Table 4:** Association of different GLP-1RAs with acute kidney injury.

Drug name	N	ROR(95%CI)	PRR(χ^2^)	IC(IC025)	EBGM(EBGM05)
Exenatide	880	0.66 (0.62-0.71)	0.66 (12.38)	-0.59	0.67 (0.63)
Liraglutide	934	1.50 (1.41-1.60)	1.49 (52.87)	0.57 (0.54)	1.49 (1.41)
Dulaglutide	546	0.70 (0.64-0.76)	0.70 (8.03)	-0.51	0.70 (0.66)
Albiglutide	62	0.45 (0.35-0.58)	0.46 (1.01)	-1.13	0.46 (0.37)
Lixisenatide	4	1.01 (0.38-2.72)	1.01 (34.75)	0.02 (0.01)	1.01 (0.44)
Semaglutide	244	1.38 (1.21-1.56)	1.37 (31.38)	0.45 (0.40)	1.37 (1.23)

N, the number of reports of GLP-1RA associated acute kidney injury; ROR, reporting odds ratio; CI, confidence interval; PRR, proportional reporting ratio; χ2, chi-squared; IC, information component; EBGM, empirical Bayes geometric mean.

### Onset time of GLP-1RA-related acute kidney injury

**Figure 2:**
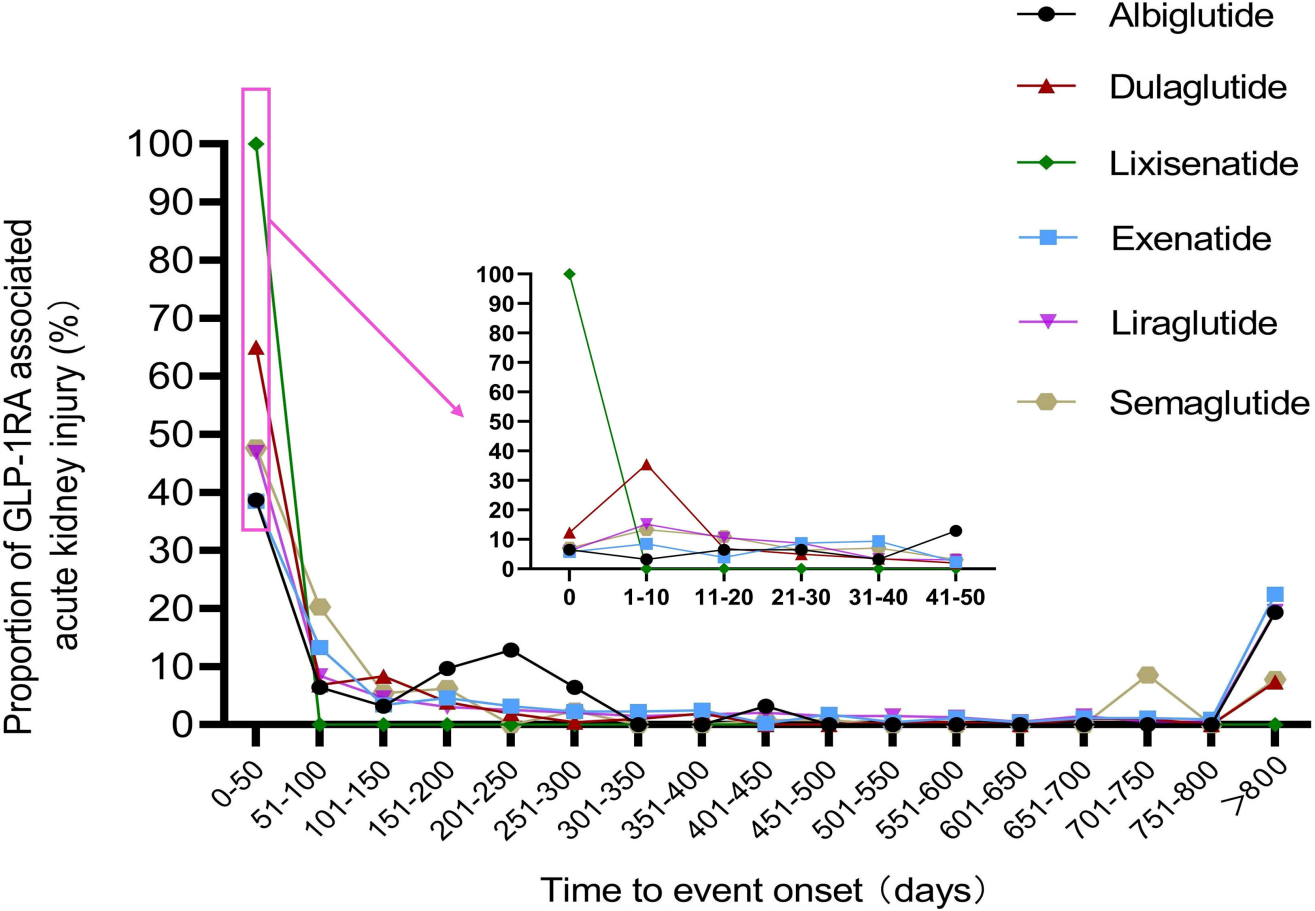
Distribution curve of the onset time of acute kidney injury caused by GLP-1RA.

Overall, the median onset of AKI with GLP-1RA was 63 days [interquartile range (IQR): 15-458.5 days]. As shown in **Figure 2**, the time of AKI caused by different GLP-1RA was calculated as follows: median time of lixisenatide was 0 days, albiglutide was 151 days [interquartile range (IQR): 41.5-252 days], exenatide was 94 days [interquartile range (IQR): 30-664.75 days], dulaglutide was 14 days [interquartile range (IQR): 6-122.5 days], liraglutide was 62 days [interquartile range (IQR): 13-510 days], and semaglutide was 51 days [interquartile range (IQR): 14.75-163.2.5 days]. Notably, all GLP-1RA (except lixisenatide) were separately reported to cause AKI on day 1 after administration. In addition, there was a significant statistical difference in the time of adverse reactions of different GLP-1RA (Kruskal Wallis test, p < 0.01). There was a difference in the time to onset of AKI between patients treated with dulaglutide and those treated with albiglutide (Mann Whitney test, p=0.003), exenatide (Mann Whitney test, p < 0.001), liraglutide (Mann Whitney test, p < 0.001) and semaglutide (Mann Whitney test, p=0.023).

### Outcome of acute kidney injury induced by GLP-1RA

**Figure 3:**
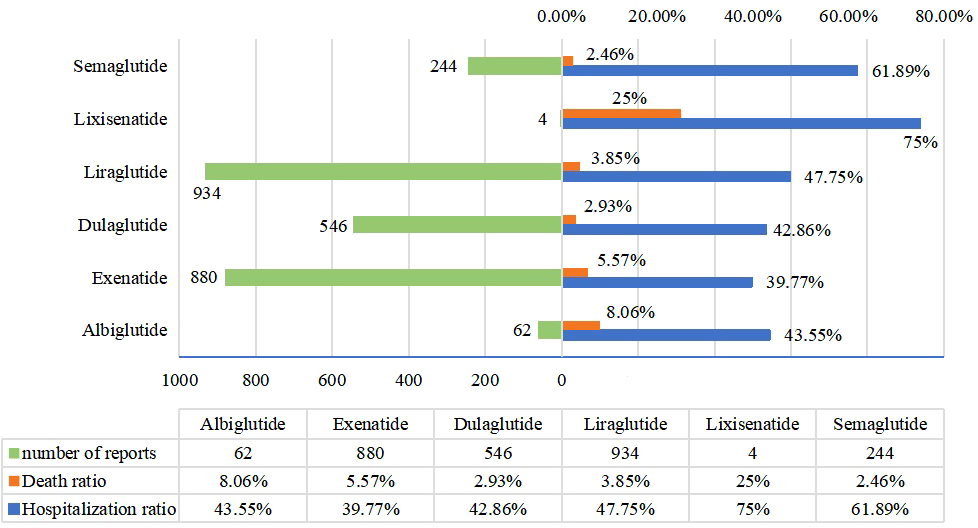
Mortality and hospitalization rate of acute kidney injury caused by GLP-1RA.

In order to analyze the outcome of adverse reactions, we calculated the mortality and hospitalization rate of AKI caused by GLP-1RA. We screened the reports with adverse reaction outcomes. The results are shown in **Figure 3**. The hospitalization rate of patients with AKI after GLP-1RA was 45.28%, while 4.23% of patients who developed AKI died. In general, AKI often indicates a poor prognosis. In this study, the mortality of lixisenatide is the highest (25%), followed by albiglutide (8.06%), exenatide (5.57%), liraglutide (3.85%), and dulaglutide (2.93%), and the lowest mortality is semaglutide (2.46%). Compared with dulaglutide(Fisher’s exact test,p=0.026) and semaglutide(Fisher’s exact test,p=0.045), the mortality of exenatide was significantly different. Interestingly, except for lixisenatide (75%), the highest hospitalization rate of patients with AKI after GLP-1RA was semaglutide (61.89%), followed by liraglutide (47.75%). There was a significant difference in hospitalization rate between semaglutide and other GLP-1RA (except lixisenatide) (p < 0.01).

## Discussion

To the best of our knowledge, this study is the first and largest to investigate the risk of AKI with different GLP-1RA using the FAERS pharmacovigilance database and to compare the onset time and outcomes of adverse reactions. According to the results, liraglutide, semaglutide, and lixisenatide were correlated with AKI and showed different clinical characteristics.

Exenatide, the first drug in GLP-1RA, was released in 2005. Since the beginning of exenatide, new drugs of the GLP-1RA family have appeared continuously, including liraglutide, dulaglutide and lixisenatide, and so on. In the clinical trial stage of these drugs, GLP-1RA showed a positive effect on the kidney. However, a meta-analysis on the clinical trial of new hypoglycemic drugs showed that the risk of AKI caused by GLP-1RA was neutral and higher than that of another hypoglycemic drug, sodium-glucose co-transporter 2 inhibitor (SGLT2i) (27).GLP-1RA has been widely used since its marketing because of its significant hypoglycemic effect and low risk of hypoglycemia, but kidney- related satefy events continue to occur. At first, several reports showed that exenatide could cause unexplained AKI, which was explained by the decrease in fluid intake and large fluid loss caused by gastrointestinal reactions (18). However, with the marketing of such drugs, such as liraglutide, semaglutide, and dulaglutide, acute kidney injury events are accompanied by GLP-1RA, and not all cases can be attributed to prerenal problems (21, 28).

Due to the low incidence rate of AKI, the above studies failed to summarize the general characteristics of adverse reactions. Compared with other studies, drug clinical trials are an essential step to confirm the effectiveness and safety of new drugs. However, due to its limitations, limited sample size, and relatively limited drug observation period, clinical trials cannot obtain definitive research conclusions. Spontaneous reporting system(SRS) is an important information source for post-marketing safety monitoring of drugs. A large number of safety data can help us judge the correlation between GLP-1RA and AKI and obtain clinical characteristics. The currently approved indications of GLP-1RA include weight management, type 2 diabetes mellitus, and reducing the risk of cardiovascular disease recurrence in adults. Attributed to their multi-organ benefits in the kidney and cardiovascular system, these drugs have also become a popular choice for adjuvant therapy in type 1 diabetes mellitus (29). At present, the treatment of type 1 diabetes mellitus is still insulin, but the long-term treatment effect of intensive insulin therapy is not ideal. Adjunctive drug therapy can complement insulin management for better glycemic control and may provide additional benefits. The clinical trial results confirm that although GLP-1RA will not reduce the HbA1c of type 1 diabetic patients, it can significantly reduce insulin consumption and help patients lose weight. (29) (which needs more clinical trials to verify). In the future, more patients may be treated with GLP-1RA as adjuvant therapy for type 1 diabetes mellitus. We think it is necessary to include the report on type 1 diabetes mellitus. Of course, patients with type 2 diabetes mellitus must be the majority of patients treated with GLP-1RA, and only a small number of patients with type 1 diabetes mellitus who are receiving cutting-edge therapies are treated with GLP-1RA. Due to the particularity of SRS, the impact of different diagnoses on safety results is weak, although the reasons for medication recorded in the report are diverse. GLP-1RA has played an increasingly important role in the treatment of diabetes (30). We think it is necessary to remind doctors to pay more attention to AKI. According to the data mined from FAERS, we can observe that the incidence rate of AKI in males is slightly higher than that in females (47.94% vs 43.48%), which may be related to the fact that the prevalence of type 2 diabetes mellitus in males is higher than that in female (31). Another study pointed out that due to gender differences, obese middle-aged men are more likely to develop insulin resistance and suffer from diabetes than women (32). Therefore, gender differences in GLP-1RA-induced AKI need to be verified by a more rigorous design. In addition, we found that adverse events were concentrated in obese patients aged 45-84 years (73.15%) or with bodyweight > 99kg (24.42%). Middle-aged and elderly people or high body weight seem to be high-risk groups for diabetes. An epidemiological study also supports this conclusion, and the prevalence of type 2 diabetes mellitus in different countries is mainly among middle-aged and elderly people (55-79 years old) (33). These conditions are also the main groups treated with GLP-1RA. GLP-1RA has a good weight loss effect, so it is mostly used for obese diabetic patients (34). Age and obesity are high-risk factors for kidney disease (35, 36). Therefore, we suggest that renal function should be assessed before treatment in these patients.

In our study, liraglutide, semaglutide, and lixisenatide were associated with AKI. Post-marketing studies of liraglutide have confirmed that the increase in serum creatinine and AKI are mostly caused by nausea, vomiting, dehydration, or the combined use of renin-angiotensin-aldosterone system inhibitors (37). Although the hypothesis of indirect kidney injury is generally accepted, there are still unexplained acute kidney diseases, such as interstitial nephritis (20), which was also confirmed by semaglutide (21).In addition, studies have confirmed that liraglutide further impairs body fluid balance by increasing renal sodium excretion (14). At present, there are few clinical trials on AKI induced by GLP-1RA. The reasons may be related to the fact that AKI is a rare adverse reaction, the observation period of clinical trials is limited, and the adverse reactions are easily covered by the rapid progress of diabetic kidney disease(DKD).In this study, the adverse signals of exenatide, dulaglutide, and albiglutide in AKI were not found, but some studies have confirmed the close connection between exenatide and AKI (38), and dulaglutide is also considered to cause a decrease in body fluid intake (14), which may be one of the reasons for AKI (28). Albiglutide has been on the market for a short time and has applied for delisting from FDA in 2018. At present, there is no evidence of its safety research. Although this study has mined risk signals, because the currently available evidence is limited, we are unable to explain the different research results of inconsistencies. However, we believe that with the deepening of the study, the emergence of more clinical trials or cohort studies can verify the risk of GLP-1RA-associated renal injury, to balance the risk and clinical benefits.

The mining methods adopted in this study have their own characteristics. ROR and PRR have similar analysis principles and good consistency of results (39). However, the accuracy of the method is excessively dependent on the number of reports of adverse reactions related to target drugs, and a small number of reports in the database will significantly reduce the accuracy of the method (40). BCPNN and MGPS have robust calculation results and strong ability to predict adverse reactions, but also have low sensitivity (41). There are differences in the number of signals generated by different analysis methods (42). Therefore, a single method is not persuasive when used for signal generation. In addition, some studies have found that although signals can be obtained by different discovery criteria, the number of signals is not consistent (42). GLP-1RA has been on the market for a relatively short time, and the composition of various adverse reactions caused by the drug is not stable, which may lead to false-negative results. In this study, four methods are used to mine the target signal and obtain stable results as far as possible, but we cannot deny the possibility of signal loss. Therefore, although exenatide, dulaglutide and albiglutide did not generate signal, in order to summarize the rule of GLP-1RA leading to AKI, we still analyzed the onset time and mortality rate of adverse reactions.

We are also very concerned about the onset time of adverse reactions. We found that the median time of AKI caused by GLP-1RA was 63 days [interquartile range (IQR): 15-458.5 days]. Drug-induced AKI has always been a topic of clinical distress because of the diversity of pathogenesis (43), which leads to the inestimable timing of its occurrence. In general, the risk of AKI after treatment can range from a few days to 1-2 years, which makes it difficult for clinicians to determine possible cause-effect relationships. We suggest that renal function indicators should be monitored regularly after treatment. Furthermore, it seems that dulaglutide is prone to AKI earlier than other GLP-1RA, which may be related to the reduction of total body fluid (14).

To further analyze the severity of AKI, we compared the hospitalization rate and mortality rate of patients with AKI after treatment with different GLP-1RA. Generally speaking, the results of AKI are not expected. Some studies have pointed out that drug-related AKI is associated with high mortality (44). Fortunately, just 4.23% of these patients died from AKI. The mortality was not particularly high, while the hospitalization rate reached 45.28%, indicating that most patients had serious symptoms though, they received attention with active treatment as a result of adverse reactions. Surprisingly, lixisenatide and albiglutide showed worrisome high mortality, which may be related to the small number of adverse reactions of the two drugs, and the accuracy of the data was affected. Furthermore, although the hospitalization rate of exenatide is not high, its mortality is statistically higher than that of dulaglutide and semaglutide. The results of several post-marketing studies have shown that AKI caused by exenatide is often unsatisfactory, with incomplete recovery of renal function or even renal failure (45). This discovery needs to arouse the attention of doctors. We should pay more attention to the use of exenatide in patients at risk of kidney disease, and strengthen the care and treatment after the occurrence of adverse reactions. On the contrary, the results of semaglutide were just the opposite. The hospitalization rate was statistically significantly higher than that of other GLP-1RA, but the mortality was low (2.46%), indicating that semaglutide had a high risk of AKI, but the severity may be relatively low.

As GLP-1RA has been increasingly widely used in the treatment of diabetes, AKI caused by GLP-1RA should be treated with caution. The results of this study can be used for doctors’ clinical decision-making. When choosing GLP-1RA as the hypoglycemic regimen, it is necessary to pay special attention to patients with high renal risk and strengthen their monitoring. In addition, according to the current study (28), as long as it is clear that AKI is caused by GLP-1RA, excluding drug-related factors, the timing of drug withdrawal is very important. Appropriate cessation of suspected drug treatment can significantly reduce the severity of patients’ diseases. During GLP-1RA treatment, attention should be paid to the relevant manifestations of AKI, such as oliguria, anuria, elevated blood potassium, azotemia, acidosis, etc.

Based on real-world data, this study uses data mining technology to obtain adverse reaction signals, which has advantages over other studies in the same field. However, we have to admit that the self-reporting database has its limitations. First of all, SRS cannot proactively collect adverse event information, but can only receive the self-reported adverse reaction data. Therefore, repeated or omitted reporting is inevitable, which will affect the results of data mining. Based on the voluntary nature of adverse event reporting, we used the FAERS database to establish associations between different GLP-1RA and AKI, but the results could not be used to compare the safety between GLP-1RA. Secondly, in the process of sorting out the data, we found that there was incomplete information in the reported cases, which directly led to the deviation of the analysis results. Finally, due to the shortcomings of the database itself, we were unable to investigate the underlying diseases of the cases, nor to obtain the original renal function of the patients, which prevented us from discovering the full range of risk factors for AKI. Despite the limitations mentioned above, the short marketing time of GLP-1RA and the limited number of reported safety events, the data mined in this study confirmed the association between GLP-1RA and AKI, providing clues for further clinical research. We will continue to explore the relationship between GLP-1RA and AKI in the next study.

## Conclusion

In this study, we mined and analyzed AKI signals of different GLP-1RA based on FAERS. What is most interesting about this study is that we found the strongest association between liraglutide with AKI. In addition, renal function should be monitored regularly from the initial stage of GLP-1RA, especially in patients at high renal risk. Other risk factors for AKI include advanced age and obesity. Our study provides a basis for sustained pharmacovigilance. We look forward to more abundant epidemiological investigations to verify the conclusions of this study.

## Data availability statement

Publicly available datasets were analyzed in this study. This data can be found here: https://www.fda.gov/drugs/questions-and-answers-fdas-adverse-event-reporting-system-faers/fda-adverse-event-reporting-system-faers-latest-quarterly-data-files.

## Author contributions

SD wrote the manuscript. SD and CS edited the manuscript. CS provided direction. All authors contributed to the article and approved the submitted version.
